# Radiation-Induced Sarcomas of the Breast: A Review of a 20-Year Single-Center Experience

**DOI:** 10.7759/cureus.38096

**Published:** 2023-04-25

**Authors:** Vanessa Di Lalla, Marwan Tolba, Farzin Khosrow-Khavar, Ayesha Baig, Carolyn Freeman, Valérie Panet-Raymond

**Affiliations:** 1 Radiation Oncology, McGill University, Montreal, CAN; 2 Radiation Oncology, McGill University Health Centre, Montreal, CAN; 3 Epidemiology and Statistics, McGill University, Montreal, CAN; 4 Pathology, McGill University, Montreal, CAN

**Keywords:** multidisciplinary management, radiation-induced sarcoma, breast, breast sarcoma, radiation therapy

## Abstract

Background

Radiation-induced sarcomas (RISs) are histologically proven sarcomas within or around a previously irradiated site, per Cahan’s criteria. RIS incidence is higher in breast cancer compared to other solid cancers and the prognosis remains poor given limited treatment options. This study aimed to review 20-year experience with RISs at a large tertiary care center.

Methodology

Using our institutional cancer registry database, we included patients meeting Cahan’s criteria diagnosed between 2000 and 2020. Patient demographics, oncologic treatment, and oncologic outcomes data were collected. Descriptive statistics were used to describe demographic data. Oncologic outcomes were assessed using the Kaplan-Meier method.

Results

A total of 19 patients were identified. The median age at RIS diagnosis was 72 years (range = 39-82 months), and the median latency period for the development of RIS was 112 months (range = 53-300 months). All patients underwent surgery, three patients received systemic therapy, and six patients received re-irradiation as salvage treatment. The median follow-up time was 31 months (range = 6-172 months) from the diagnosis of RIS. Overall, five patients had local recurrence, and one patient developed distant metastases. The median time to progression was seven months (range = 4-14 months). The progression-free survival (95% confidence interval (CI)) at two years was 56.1% (37.4-84.4%). At follow-up two years after the diagnosis of sarcoma, the overall survival (95% CI) was 88.9% (75.5-100%).

Conclusions

While breast RIS remains rare, when managed in a large tertiary care center, overall survival outcomes appear favorable. A significant proportion of patients recur locally after maximal treatment and require salvage therapy to improve outcomes. These patients should be managed in high-volume centers where multidisciplinary expertise is available.

## Introduction

Despite the widespread use of radiotherapy in the management of different malignancies, the mechanism of radiation-induced carcinogenesis remains poorly understood, with hypotheses centered around the direct ionizing effect of radiation causing DNA damage, the release of reactive oxygen species, or bystander effects leading to the release of inflammatory mediators [1]. Radiation-induced sarcomas (RISs) are rare malignancies that comprise only 2.5-5.5% of all sarcomas [2]. RISs were first defined by Cahan et al. in 1948, and diagnosis relies on temporal and spatial relation to radiotherapy and a prolonged latency period [3].

Prognosis is generally poor, with five-year overall survival estimates in the literature varying widely from 17% to 58% [2,4-9] compared with 54% to 76% in sporadic sarcomas [2]. Poor outcomes are likely partly due to limited management options in the context of previous treatment as well as local and distant disease aggressiveness [2]. Complete surgical resection is associated with improved disease-specific survival but is only achieved in a minority of patients [10].

Patients who receive radiotherapy for primary breast cancer are known to have a higher incidence of RISs compared to other primary solid cancers [11]. A recent review of Surveillance, Epidemiology, and End Results (SEER) data found an incidence of 0.02% in a population of breast cancer patients diagnosed from 1973 to 2013 [11]. This is lower than older studies which found the incidence ranging from 0.03% to 0.2% [12-15]. With improvements in breast cancer survival, it is important to note that RISs typically occur 10 years following breast irradiation, but this latency period can be as long as 20 years [16] or as short as six months [17]. Radiation-induced cutaneous angiosarcomas of the breast have a shorter latency period of four years [16].

Given the large number of breast cancer patients diagnosed and treated every year, this study aimed to review the incidence, risk factors, management, and subsequent oncologic outcomes of RISs of the breast using the 20-year experience at the McGill University Health Centre (MUHC), a large tertiary care center.

This article was previously presented as a meeting abstract at the 2022 Canadian Association of Radiation Oncology (CARO) Annual Scientific Meeting on September 29, 2022.

This article was previously posted to the Research Square preprint server on August 9, 2022.

## Materials and methods

Data collection

After obtaining research ethics board approval, we retrospectively reviewed our institutional cancer registry database to identify all patients with histologically confirmed sarcoma of the breast diagnosed between 2000 and 2020. This includes patients diagnosed within our center as well as patient referrals. We only included patients who had a primary breast cancer diagnosis. To diagnose RIS, we used Cahan’s criteria, which include (1) a history of radiation exposure before the development of sarcoma, (2) sarcoma within or around the field of radiation, (3) a prolonged latency period, and (4) histological confirmation of diagnosis that differs from the primary cancer that was treated [3]. Patients who did not meet these criteria were excluded. Using our electronic medical records, we collected patient demographic data, oncologic treatment data for both the primary breast cancer and the secondary sarcoma, as well as oncologic outcomes.

Statistical analysis

We used descriptive statistics to summarize patient demographic data including mean, median, and standard deviation for continuous variables and frequencies and proportions for categorical variables. Oncologic outcomes including overall survival probability and progression-free survival (PFS) probability, with endpoints such as recurrence or death, and corresponding 95% confidence intervals (CIs) were generated using the Kaplan-Meier method [18].

## Results

From 2000 to 2020, approximately 8,700 patients were treated with radiotherapy for breast cancer in our institution. Including three patients referred from outside centers, we identified a total of 19 patients with breast RISs: 11 angiosarcomas (57.9%), three osteosarcomas (15.8%), two carcinosarcomas (10.5%), two undifferentiated pleomorphic sarcomas (10.5%), and one high-grade leiomyosarcoma (5.3%). The median age at diagnosis of primary breast cancer was 59 years (range = 29-78 years, mean = 56 years). In total, 18 patients underwent breast-conserving surgery (94.7%), and one patient underwent total mastectomy as their primary treatment. All patients received radiotherapy. Seven patients received adjuvant chemotherapy (36.8%) and eight patients did not (42.1%), while for four patients chemotherapy status was unknown (21.1%). A total of 13 patients had hormone receptor-positive breast cancer (68.4%) and received endocrine therapy. Data on radiotherapy volumes, dose, and fractionation were missing in three patients who were treated at outside institutions (15.8%). Radiotherapy treatments were given as three-dimensional conformal radiotherapy and included breast or chest wall only or locoregional radiotherapy including axillary supraclavicular plus or minus internal mammary lymph node irradiation. Four (21.1%) patients had a primary breast boost to their surgical cavity. Only one patient had a known genetic cause for breast cancer (TP53 mutation). Patient and primary disease characteristics are summarized in Table 1.

**Table 1:** Patient characteristics and primary disease and treatment. *: Four patients for whom chemotherapy status is unknown are included in the surgery alone or surgery and endocrine therapy categories.

		n = 19 (% total)
Age at primary diagnosis (years)	≤40	3 (15.8%)
	41–50	3 (15.8%)
	51–70	10 (52.6%)
	≥70	3 (15.8%)
T stage	T1	7 (36.8%)
	T2 or more	5 (26.3%)
	Unknown	7 (36.8%)
Nodal status	Node negative	9 (47.3%)
	Node positive	3 (15.8%)
	Unknown	7 (36.8%)
Primary treatment other than radiotherapy*	Surgery alone	4 (21.1%)
	Surgery and chemotherapy	2 (10.5%)
	Surgery and endocrine therapy	9 (47.3%)
	Surgery, endocrine, and chemotherapy	4 (21.1%)
Radiotherapy received	Breast/chest wall only	14 (73.7%)
	Locoregional	2 (10.5%)
	Unknown	3 (15.8%)
Radiotherapy boost received	Yes	4 (21.1%)
	No	12 (63.2%)
	Unknown	3 (15.8%)

The median age at diagnosis of secondary sarcoma was 72 years (range = 39-82 years, mean = 67 years). Taken from the end of previous radiation treatment, the median latency period to develop RIS was 112 months (range = 53-300 months, mean = 120 months). All patients underwent surgery at the time of sarcoma diagnosis, with the majority of patients (73.6%) undergoing total mastectomy. There were three patients who had positive margins (15.8%), one patient with margin status not reported (5.3%), and the remainder had negative margins (78.9%). Among the patients with negative margins, only two had wide margins greater than 1 cm (10.5%). Six (31.6%) patients received re-irradiation. Only three (15.8%) patients received systemic therapy. At a median follow-up of 31 months (range = 6-172 months, mean = 48 months) from diagnosis of RIS, four (21.1%) patients were deceased and two (10.5%) patients were lost to follow-up. RIS diagnosis and treatment data are summarized in Table 2.

**Table 2:** RIS diagnosis and treatment characteristics. RIS: radiation-induced sarcoma; UPS: undifferentiated pleomorphic sarcoma; LMS: leiomyosarcoma

		n = 19 (% total)
Age at RIS diagnosis (years)	≤40	1 (5.3%)
	41–50	2 (10.5%)
	51–70	6 (31.6%)
	≥70	10 (52.6%)
Histology	Angiosarcoma	11 (57.9%)
	Osteosarcoma	3 (15.8%)
	UPS	2 (10.5%)
	High-grade LMS	1 (5.3%)
Treatment received	Surgery alone	11 (57.9%)
	Surgery and chemotherapy	2 (10.5%)
	Surgery and radiotherapy	5 (26.3%)
	Surgery, radiotherapy, and chemotherapy	1 (5.3%)
Surgical margin status	Positive	3 (15.8%)
	Negative	15 (78.9%)
	Unknown	1 (5.3%)
Status at analysis	Alive	13 (68.4%)
	Diseased	4 (21.1%)
	Unknown	2 (10.5%)

**Figure 1:**
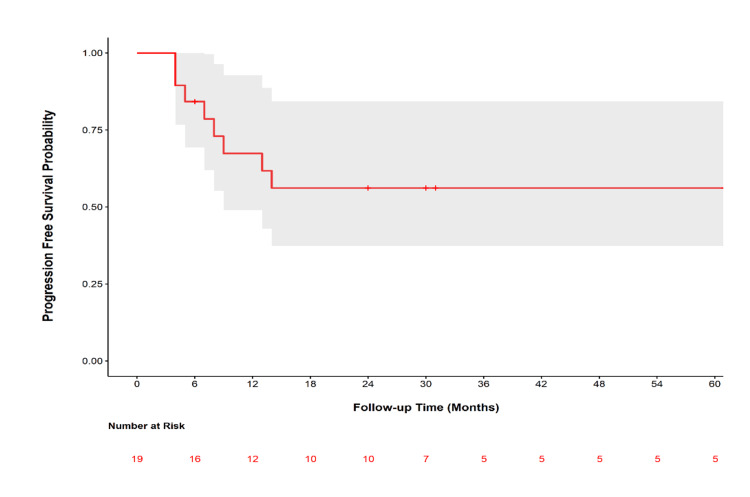
Kaplan-Meier estimates of progression-free survival probability. *: The patients were censored at the following months: six, 24, 24, 24, 30, and 31 (up to five years). Three patients were censored at 24 months and two patients experienced study outcomes (one recurrence at the site of sarcoma and one bone metastasis) at month four.

There were six (31.6%) patients who had a documented PFS event: five patients had recurrence at the site of sarcoma, and one patient developed distant metastases. For patients who had progression, the median time to progression was seven months (range = 4-14 months). The PFS (95% CI) at two years was 56.1% (range = 37.4-84.4%). At the two-year follow-up after sarcoma diagnosis, two patients had died from their disease, resulting in a disease-specific overall survival (95% CI) of 88.9% (range = 75.5-100%). The other disease-related death occurred at 40 months. The fourth death was due to a third (pancreatic) cancer. The Kaplan-Meier curve for PFS is shown in Figure 1.

## Discussion

Our center is a large tertiary care center that treated approximately 8,700 breast cancer patients with radiotherapy in the 20-year period included in this study. We are also a referral center for sarcoma patients diagnosed at outside centers. We are specialized in sarcoma management, employing a multidisciplinary approach that includes surgical oncology, medical oncology, and radiation oncology. To our knowledge, this is one of the first studies exploring RIS of the breast in a cohort of patients diagnosed and treated with modern techniques between 2000 and 2020.

In our study, we found 19 patients with RISs of the breast over the 20-year period. Taghian et al. examined 7,000 breast cancer patients treated between 1954 and 1983 in France [14]. They found 11 patients who developed secondary sarcoma, resulting in a cumulative incidence of 0.2% at 10 years. However, noticeably different radiotherapy techniques were used in this historic cohort, as some patients received total body irradiation in addition to breast radiotherapy, resulting in a higher total radiotherapy dose [14]. They reported a median survival of 2.4 years, with 55% of patients alive at two years [14]. In comparison, 88.9% of our patient population was alive at two years. In their 2002 publication, Yap et al. used SEER data to identify breast cancer patients who underwent radiotherapy from 1973 to 1997 (n = 275,000) [15]. The cumulative incidence of sarcoma in patients who received radiotherapy was 0.32% at 15 years compared to 0.23% in patients who did not receive radiotherapy [15]. They reported a five-year overall survival of 27.5%, with a median survival of 2.3 years [15]. In a more recent analysis of the SEER database, Snow et al. found the incidence of RIS of the breast to be 0.02% at a median follow-up of 9.6 years [11]. A major limitation of SEER data is that it does not report data on radiation treatment or systemic treatment received [11,15]. The SEER data analysis also excluded patients for whom data were missing, therefore, likely underestimating the true incidence.

While less than 10% of secondary malignancies are radiation-induced, the relative risk of developing RIS varies by treatment site and was found to be correlated with a higher dose, younger age, and increased time since diagnosis in one review [19]. The dose is an important factor, with risk increasing linearly after 40 Gy [19]. The risk of sarcoma was 30.6 times higher for doses more than 44 Gy compared to doses less than 15 Gy in breast cancer patients in one study [20]. All of the patients included in our analysis in whom radiotherapy dose was known received doses of at least 40 Gy, consistent with standards of care for breast radiotherapy.

Breast cancer patients were found to have the highest incidence of RIS compared to other primary solid cancers in a recent review of SEER data [11]. Hereditary breast cancer syndromes (Li Fraumeni, retinoblastoma, Nijmegen breakage syndrome) and BRCA are thought to play a role [21]. Kadouri et al. reviewed 473 *BRCA *and *p53 *mutation carriers and found the rate of RIS to be 0.43% (n = seven women), but the overall rate is still low and not significantly different compared to all breast cancer patients with RISs reported in other studies [21]. Schlosser et al. retrospectively reviewed 230 women with *BRCA *mutations who underwent radiotherapy for breast cancer and found the incidence of secondary malignancies to be 0.32 per 1,000 women-years [22]. However, of the six women who developed secondary malignancies, none were RIS [22]. In our study, we identified one patient with a *TP53 *mutation. However, it is difficult to draw conclusions on hereditary breast cancer and the incidence of RIS given the rarity of both.

While this study yields important insights into the incidence and outcomes of RIS of the breast, it has several limitations. First, inherent to its retrospective nature, data were missing, for example, pertaining to previous radiation treatment details for patients who were treated at outside institutions. Two patients were lost to follow-up, further limiting our data analysis. Furthermore, we included data from electronic health records only and did not have access to paper charts which may have been used in the early 2000s. Given that this is single institutional data and the incidence of breast RIS is rare, the total number of patients included in our analysis is small. This small number of patients renders it difficult to draw conclusions on risk factors associated with poorer outcomes.

## Conclusions

Our overall survival was favorable, with a two-year disease-specific survival of 88.9%. Our PFS of 56.1% at two years illustrates that a large proportion of patients recur locally or distantly despite aggressive multimodality treatment, suggesting that longer follow-up time is needed for survival data maturation.

We show that overall survival and PFS for RIS of the breast appear favorable when salvage is possible and patients are managed with a multimodality approach. Patients should ideally be managed in large centers with multidisciplinary expertise. Further studies should evaluate multi-institutional data because the rarity of RIS renders it difficult to draw conclusions on risk factors.
